# Using Multistate Models and Qualitative Interviews to Comprehensively Characterize Substance Use Disorder Care Transitions in a US Health Care System: Protocol for a Mixed-Methods Study

**DOI:** 10.2196/93043

**Published:** 2026-05-28

**Authors:** Joseph E Glass, Edwin S Wong, Tara C Beatty, Jennifer F Bobb, Randall Brown, Paul Fishman, Abisola Idu, Jubi Y L Lin, Theresa E Matson, Lorella Palazzo, Arvind Ramaprasan, Zelda B Zabinsky, Noorie Hyun

**Affiliations:** 1 Lighthouse Institute, Chestnut Health Systems, 1255 Pearl Street, Suite 201, Eugene, OR, 97401, United States, 1 3094517837; 2 Kaiser Permanente Washington Health Research Institute, Seattle, WA, United States; 3 VA Puget Sound Health Care System, Seattle, WA, United States; 4 Department of Health Systems and Population Health, University of Washington, Seattle, WA, United States; 5 Department of Biostatistics, University of Washington, Seattle, WA, United States; 6 Department of Family Medicine and Community Health, School of Medicine and Public Health, University of Wisconsin–Madison, Madison, WI, United States; 7 Department of General Internal Medicine, University of Washington, Seattle, WA, United States; 8 Industrial and Systems Engineering, University of Washington, Seattle, WA, United States

**Keywords:** electronic health records, substance use, care trajectories, treatment episodes, multistate modeling

## Abstract

**Background:**

Many substance use disorder (SUD) care pathways exist in health care systems. However, patients with SUD often report poor care experiences, particularly regarding timely follow-up, clinician and general satisfaction ratings, and care communication. As SUD care pathways involve transitions across clinicians and venues of care, adequate treatment requires coordination across clinicians and settings to ensure unmet needs are addressed, and appropriate SUD care is delivered. Surprisingly little is known about the real-world care pathways patients engage in once identified as having SUD.

**Objective:**

This study protocol describes research that will comprehensively characterize the pathways of care used by those who obtain care for SUD and compare the quality and outcomes associated with these care pathways. Specific aims are to (1) apply multistate models (MSM) to characterize the spectrum of care transitions among patients with SUD within a large integrated health system, (2) generate data-driven insights to improve care delivery for SUD using estimated MSMs, and (3) observe and explore patient and clinician experiences with care transitions across common care pathways using qualitative methods.

**Methods:**

Quantitative data sources will include electronic health care records, insurance claims, self-reported measures of substance use and SUD symptoms, and death data from an integrated health care system in Washington State. To identify care pathways, we will apply continuous time, multistate modeling methods to empirically observe the longitudinal course of SUD care transitions undertaken by patients over time; each “state” or occurrence of care will be characterized by the intervention received (eg, evaluation or assessment, behavioral treatment or counseling, and pharmacotherapy) and setting of care (eg, outpatient, intensive outpatient, or inpatient or residential). The estimated parameters from the fitted MSMs will be transformed or interpreted to characterize SUD care quality, such as wait times for SUD visits, and receiving an adequate psychotherapy dose. To compare outcomes associated with care pathways, we will examine terminal states, including death and loss to follow-up. Using a mixed methods design, we will sample and interview patients engaged in empirically derived pathways to understand their care experiences, observe and interview clinicians to elucidate health system factors that impact SUD care transitions, and integrate qualitative and quantitative findings using joint displays.

**Results:**

This study was funded in June 2025 and received institutional review board approval on July 17, 2025. We expect preliminary data collection for quantitative analyses to be complete by May 2026, and final data collection will be complete by November 2027. We expect qualitative data collection completion by November 2029.

**Conclusions:**

This exploratory study will identify and compare the quality and outcomes associated with common pathways that patients take when they obtain treatment for SUD and provide decision-makers with information on how to better organize SUD care delivery.

## Introduction

Unhealthy substance use, including alcohol, cannabis, and prescription or illegal drugs, is a critical problem for the US health care system. Approximately 30% of people in the United States report unhealthy alcohol use [1,2] and 25% report unhealthy drug use [3]. Substance use contributes to disability and societal costs [4-8]. Substance use disorder (SUD) is a pattern of substance use that causes clinically significant impairment or distress [9,10]. High numbers of deaths driven by overdose from synthetic drugs and counterfeit medications [11], as well as chronic medical complications due to long-term use [12], underscore the urgency of this problem.

Well-defined treatment pathways for SUD exist, and include screening and assessment, behavioral counseling, pharmacological interventions, and/or referral to specialty treatment [13-15]. However, patients with SUD experience substantial challenges in receiving recommended care. Health systems struggle with long wait times that hinder patients’ ability to access SUD care. Clinicians cite challenges in facilitating access to appropriate SUD care and may lack knowledge of available care options. Care coordination is critical for managing patients with SUD, given the wide spectrum of SUD care options, and care is often chronic and prolonged, yet research has found that care is poorly coordinated [16]. Collectively, these challenges result in lengthy follow-up, poorer care communication, and lower clinician ratings [17], and are among the reasons why ≈30% of patients drop out of SUD treatment, compromising treatment effectiveness [18,19]. New solutions are needed to increase access, continuity, and quality of SUD care in health systems.

Studies of SUD care improvements require innovative approaches that consider the dynamic, longitudinal nature of treatment required to address SUD, which has been characterized as a chronic health condition [20,21]. The National Academy of Medicine has encouraged the application of systems approaches to improving health care [22]. As an approach for handling multiple events and time-to-event data within patients, multistate approaches conceptualize health care events and outcomes as states and capture the full sequence of a patient’s care and timing of transitions between states during a care episode [23]. From a multistate modeling perspective, care pathways are defined as the longitudinal sequence of SUD visits that begin where patients were identified (eg, primary care or emergency department [ED]), transition between SUD care types (eg, evaluation and assessment, behavioral treatment and counseling, and pharmacotherapy) [24-26] and settings visited (eg, outpatient, intensive outpatient, or inpatient or residential settings) [27,28], and end in terminal events, such as ceasing SUD care or death. These models capture the dynamics of how patients move through the health system and the probabilities or “intensities” of transitions between states. As a result, multistate approaches have the potential to generate meaningful insights that health systems can use to better organize care delivery for SUD.

This paper presents the protocol for a retrospective cohort study that will apply a multistate approach to nearly 10 years of data from the Kaiser Permanente Washington (KPWA) electronic health record (EHR). This includes health care encounters and patient-reported SUD outcome data from tens of thousands of patients. We will link EHR data to health insurance claims and Washington State death data. This will support a comprehensive exploratory analysis of SUD care transitions that will inform strategies to improve access, continuity, and outcomes in SUD treatment. It also describes our plans to use qualitative research methods to complement the quantitative findings, providing a mixed-methods approach to generate data- and human-driven insights for improving SUD care.

Regarding aim 1, apply multistate models (MSMs) to comprehensively characterize care transitions across the full spectrum of SUD care among patients with alcohol, cannabis, opioid, stimulant, and other SUD. Models will track movement between care states—defined by the SUD care type (eg, pharmacotherapy, behavioral treatment, and counseling) and setting—and characterize the transition probability and time that it takes to move between care states and terminal states.

Regarding aim 2, generate data-driven insights to improve care delivery for SUD using estimated MSMs. We will calculate a broad set of estimates to address critical questions such as the identification of common sequences of care that occur with greater likelihood, the likelihood of guideline-concordant care (eg, adequate doses of services over time), and the likelihood of inefficient care sequences (eg, repeated transitions between inpatient and outpatient states).

Regarding aim 3, observe and explore patient and clinician experiences with care transitions across common care pathways using qualitative research methods. Through interviews and direct observations, we will examine how individuals navigate SUD care, the factors influencing care decisions, and the systemic barriers or facilitators encountered. This aim will be guided by the Chronic Care Model [29] and will integrate insights from both patients and clinicians.

## Methods

### Study Design and Setting

The quantitative component of this study is a retrospective longitudinal observational analysis of data collected during routine care within KPWA, a large nonprofit integrated health care and insurance coverage system in the Pacific Northwest serving over 700,000 individuals across Washington State [30]. The qualitative component consists of a prospective observational study of patient visits and an interview-based study involving KPWA patients and health care staff from KPWA and partnering organizations.

KPWA operates in more than 46 clinical locations, including primary care clinics, specialty centers, urgent care facilities, and EDs. It also maintains a contracted network for services such as specialty addiction treatment and hospital care. Primary care in KPWA is delivered through team-based models involving medical assistants, nurses, and primary care providers [31]. KPWA provides a full spectrum of services for identifying and treating SUD [32-34]. All adult group practice patients are screened annually for substance use and further assessed using standardized tools if they screen positive. Treatment options include brief counseling, pharmacotherapy, and referrals to detoxification services, which may be delivered in either inpatient or outpatient settings. A mental health access center serves as a centralized hub for coordinating referrals and authorizations for specialty addiction care [33]. Most specialty SUD treatment is provided by external treatment programs via a large, contracted care network.

### Ethical Considerations

The Kaiser Permanente (KP) Institutional Review Board granted ethical approvals for the aims 1 and 2 activities. Waivers of consent and HIPAA (Health Insurance Portability and Accountability Act) Privacy Rule Authorization were granted to access, collect, and use data from secondary data sources for this research under 45 Code of Federal Regulations (CFR) 46.116(d) and 45 CFR 164.512(i). This study was deemed to have no more than minimal risks, and it would not be otherwise feasible to conduct this study without these waivers. The qualitative data collection will require informed consent and waivers of consent and will be submitted for KP Institutional Review Board review and approval before execution of aim 3. We will recruit patients via multiple modes (phone, email, or mailed letter with a US $2 preincentive) [35] to invite them to participate in the interviews. Clinician and patient participants will receive US $75 for their time.

### Quantitative Study Population

This study’s cohort will include patients who receive care for SUD. Eligible patients are adults aged 18 years and older with an incident SUD identified between January 1, 2017, and May 31, 2025. We will include both group practice enrollees, who receive most of their care from internal KPWA-owned and operated clinics, and KPWA health insurance plan enrollees who primarily receive care outside the internal delivery system (IDS). Incident SUD is defined as meeting any of the following criteria at a clinical encounter with no prior SUD indicators in the past 6 months: (1) a newly documented SUD diagnosis, or (2) two or more *DSM-5* (*Diagnostic and Statistical Manual of Mental Disorders, Fifth Edition*) symptoms reported on standardized patient symptom checklists. The index date will be the date of the qualifying incident SUD event. The 6-month “washout period” is used to define new treatment, modeled on National Committee on Quality Assurance definitions [36], but we may explore the sensitivity of sample definitions using other time frames, such as 60 days [37]. For patients who have been enrolled in KPWA for less than the length of the washout period, we will consider their first SUD event to be an incident SUD event. Provisionally, multiple qualifying incident SUD events from the same patient will be included in the analysis (see details in Statistical Analyses). Patients will be excluded from the analyses if they have opted out of research participation with the health plan (≈0.2%).

The analytic sample will be split into distinct study subcohorts comprising patients with incident SUD identified in the specific clinical settings. We anticipate using five subcohorts, including: (1) primary care, (2) urgent care, (3) ED or hospital, (4) behavioral health, and (5) all other settings. These locations are identified based on EHR and insurance claims data that code clinic departments, places of service, encounter types, and SUD diagnosis data available in each location (Table 1). Once patients are identified as having an incident SUD and meeting the inclusion criteria described above, they enter the initial state of the MSM.

**Table 1.** Study subcohorts defined by the clinical setting in which an incident substance use disorder event occurs. Incident SUD^a^ events are identified for adult patients aged 18 years and older and are categorized into 1 of the 5 subcohorts in this table.

Cohort	Location description	Specification of the triggering event
1	Primary care	Documentation of an SUD diagnosis OR 2+ SUD symptoms as part of follow-up assessment after a positive population-based SUD screen in specified setting
2	Urgent care	Documentation of an SUD diagnosis OR 2+ SUD symptoms as part of follow-up assessment after a positive population-based SUD screen in a specified setting
3	Behavioral health	Documentation of an SUD diagnosis OR 2+ SUD symptoms as part of follow-up assessment after a positive population-based SUD screen in a specified setting
4	All other settings	Documentation of an SUD diagnosis OR 2+ SUD symptoms as part of follow-up assessment after a positive population-based SUD screen in a specified setting
5	Emergency department or hospital	Documentation of an SUD diagnosis in the specified setting

a SUD: substance use disorder.

### Quantitative Data Sources

Data for this study will be drawn from KPWA’s EHR, health insurance claims, membership enrollment records, and death data, as well as additional cause of death data collected from Washington State. KPWA EHR data provide comprehensive encounter-level records on all health services received by members. These data include information on all in-person and virtual encounters, including telephone visits and secure messages. This information is primarily captured through real-time utilization and clinical information abstracted from the EpicCare EHR. Data entered into the EHR are maintained in a relational database that allows linkages across individuals, encounters, and health measures. Membership enrollment records document individuals’ enrollment history, source of insurance, benefit design, demographic information, and medical bill financial assistance requests. Health care claims submitted by contract providers and facilities will be used to capture services not provided within KPWA’s IDS. For instance, all ED care is provided outside of the IDS. KPWA obtains nearly 99% complete data of externally provided services within 6 months of delivery. From Washington State, we will obtain death data containing causes and dates of death, provided that these events occur in the state of Washington [38]. The publicly available social determinants of health (SDOH) Database created by the Agency for Healthcare Research and Quality (AHRQ) will be used to ascertain socioeconomic characteristics (eg, per capita income and unemployment rate) and health care resources (eg, number of physicians and number of hospital beds) in patients’ residence county.

### MSM Setup

Data required for fitting MSMs include care occurrences and corresponding event times to characterize state occupancy and transitions over time, along with time-invariant and time-varying covariates. SUD-related medical conditions will be incorporated as covariates when they are expected to influence care type or care setting. Additional details are provided in the analysis section. The time scale for the MSMs is continuous calendar time, with time since entry into each state reset to 0 (“clock reset”) at state entry for modeling transition intensities.

### States for the MSMs

States will capture 3 types of events related to SUD care: initial states, transient states, and terminal states. Defining states requires a balance of specificity to produce actionable information, and parsimony to allow for an estimable statistical model with accurate estimates and smaller SEs.

Initial states in the MSMs represent the clinical settings that triggered an incident event at the start of an SUD care episode, corresponding to the 5 subcohorts in Table 1.

There are 3 types of transient states. The first type is care states, which include different types of care provided in the health care system to treat the SUD, defined by combinations of setting and service type (Table 2). We opted to define 15 preliminary SUD care states based on a combination of 3 settings and 5 service types. The second type of transient state is temporary discontinuation of care. Entry into the temporary discontinuation care state is triggered using a clinically meaningful threshold for time elapsed between SUD treatment visits. The last transient state is SUD remission, which represents an improvement in the patients’ disease.

**Table 2.** Transient states and terminal states are represented in the multistate models.

Category	States included	Description
Transient states	A total of 17 transient states include 15 care states derived from crossing 5 service types with 3 care settings; temporary discontinuation of SUD^a^ care; and remission of use disorder.	Care states Service types Evaluation and assessment Behavioral treatment and counseling Prevention, education, and case management Pharmacotherapy Other service Settings Outpatient Intensive outpatient Inpatient or residential Other transient states Discontinuation of SUD care A lack of SUD care over a specified period^b^ Remission of use disorder Low risk score on alcohol and substance use screen; clinically meaningful reduction on screening scores or <2 symptoms on *DSM-5*^c^ checklist; SUD remission diagnosis
Terminal states	4 states representing the end of a care episode	Death, derived from dates in health care enrollment records or Washington State death data Loss to follow-up, defined as at least 31 days without KPWA insurance coverages Ceased SUD care (no medication fills or services with a qualifying SUD diagnosis for more than 194 days Administrative censoring (in the middle of a care episode when data collection ends)

a SUD: substance use disorder.

b Temporary discontinuation will be defined separately for each care state using an appropriate threshold for time elapsed between visits that reflects acceptable gaps in care for that specific state. All of these periods will be shorter than 194 days to distinguish temporary discontinuation from ceasing substance use disorder care.

c *DSM-5*: *Diagnostic and Statistical Manual of Mental Disorders, Fifth Edition*.

Terminal states indicate the end of an SUD care episode (Table 2), such as ceasing SUD care.

### Data Used to Construct States

#### Health Care Encounter Data

We will use health care encounter data drawn from KPWA EHR data and health care insurance claims to construct initial states, transient states, and terminal states. Data elements include *ICD-10* (*International Classification of Diseases, Tenth Revision, Clinical Modification*) diagnosis codes, health care service procedure codes, encounter type classifications (eg, inpatient, ambulatory, virtual visit, or ED), encounter subtypes (eg, urgent care), departments (eg, mental health), clinical specialty (eg, mental health, social worker, or psychiatry), revenue codes, and place of service codes. Visit timestamps are used in models to determine follow-up time and transition times.

#### Substance Use Data

Patient self-reported substance use frequency, patient self-reported SUD symptoms, and clinician-documented SUD diagnoses will be used to construct states. In the creation of initial states, diagnosis codes, service procedure codes, and revenue codes help determine whether a given health care visit was related to SUD. For instance, some initial states are defined by a new clinician-documented SUD diagnosis, while others are also defined to capture clinical workflows where patients are first identified as having substance use by the nature of a health screening (eg, substance use screening in primary care). Care states require an accompanying clinician-documented SUD diagnosis, unless the care state involves a procedure or medication that is solely indicated for SUD (eg, outpatient addiction treatment or disulfiram medication). The SUD remission state is captured using self-reported substance use frequency and SUD symptoms, and clinician-reported SUD remission diagnoses.

To obtain patient self-reported substance use frequency data, clinicians are prompted by the EHR to conduct annual screening with patients in primary care. During in-person visits, screenings are administered on paper or tablet by medical assistants, then entered into the EHR. In telephone or video visits, patients complete a questionnaire via an online patient portal. Validated substance use screening measures assess past-year alcohol, cannabis, and other drug use [32,39,40]. Patients with screening scores that are deemed to be at high risk for SUD are asked to complete a follow-up assessment for symptoms of SUD using the Alcohol Symptom Checklist and the Substance Use Symptom Checklist, respectively. These 11-item checklists capture symptoms of SUD that align with the 11 *DSM-5* diagnostic criteria [41]; the cutpoint of 2 or more symptoms is consistent with SUD. The checklists have demonstrated strong psychometric properties [42-44].

#### Clinician-Documented SUD Diagnoses

SUD diagnoses include clinically documented *ICD-10* diagnoses for alcohol, cannabis, opioid, stimulant, sedative, and other drug use disorder (eg, inhalants or hallucinogens), which are recorded in the EHR and claims data when clinicians conduct visits. SUD diagnoses will include those identified in the alcohol, opioid, and other drug “abuse and dependence” value sets from the HEDIS (Healthcare Effectiveness Data and Information Set) developed by the National Committee for Quality Assurance [45,46].

#### Death Data

Death, classified as a terminal state in the MSM, will be ascertained from the Washington State Department of Health death data. These data are available for all patients, regardless of whether they remain enrolled in the health system. Following standard practice [47], death data are triangulated from several data sources. Reliability of death date (excellent, fair, or poor) is determined based on the source, the number of reporting sources, and the strength of the match in the matching process (eg, no discrepancies in identifiers).

### Study Outcomes

This study has 2 types of outcome assessments: outcomes associated with terminal states, such as the percentage of patients or time that it takes to get to a terminal state, and outcomes associated with pathways, such as achievement of guideline-concordant care (Table 2, Multimedia Appendix 1). Terminal states are explicitly modeled as states in MSMs, whereas the probability of receiving guideline-concordant care is derived from estimated transition probabilities (see subsection Aim 2. Generate Data-Driven Insights to Improve Care Delivery Using Estimated MSMs under the Statistical Analysis section).

### Covariates

#### Sociodemographic Characteristics

Patient sociodemographic and clinical characteristics include both time-invariant and time-varying measures and will be used to describe the analytic sample. In addition, they may be used as covariates in MSMs. Planned demographic characteristics include age, race or ethnicity, sex, and insurance history (eg, Medicaid, Medicare, employer-based, or individual or family plans purchased through WA state’s health plan marketplace). Demographics are typically collected at enrollment and confirmed or updated at health care visits over time [48]. Socioeconomic status will be approximated from census block-level measures from the AHRQ SDOH Database [49]. Additionally, clinician-documented housing instability *ICD* (*International Classification of Diseases*) codes are from patients’ EHR data [50], and patients’ inability to pay for care is measured by a recent request to the KP Medical Financial Assistance program for low-income or uninsured individuals [51]. Medical morbidity [52,53], depression [54], anxiety [55], and serious mental illness [56] will be defined by *ICD* codes. AHRQ SDOH database measures will be linked to patient-level records at the county level based on patient addresses.

#### SUD-Related Health Conditions

SUD-related health conditions will be used to describe the analytic sample and as time-varying covariates in the MSMs. These include documented diagnoses for health conditions in which substance use is always the cause (eg, alcoholic gastritis, alcohol neuropathy, accidental drug poisoning, and nonfatal alcohol and illicit drug overdoses). These are measured using *ICD* codes specified by the Centers for Disease Control and Prevention’s Alcohol-Related Disease Impact codes [46,57] and the HEDIS diagnosis grouping of “unintentional drug overdose.”

### Statistical Analyses

#### Aim 1: Apply MSMs to Comprehensively Characterize SUD Care Transitions

Separate MSMs will be run for each of the 5 subcohorts specified in Table 1. The 5 subcohorts are mutually exclusive. All prespecified states that are anticipated will be considered. States may be collapsed to address states with a small number of events, and new states may emerge if they occur with sufficient frequency (eg, concurrent receipt of pharmacotherapy and behavioral treatment and counseling). When collapsing or expanding states, we will strike a balance between clinical interpretability and reliable parameter estimation. As a general guideline, ≥40‐50 transition events from *S*_*j*_ to *S_j_* per covariate helps ensure stable hazard ratio estimates and corresponding SEs, but if collapsing states compromises the clinical interpretability of the results, we will adopt a more permissive threshold of at least 10 transition events per covariate for modeling [58]. This criterion will first be applied to define states; specifically, a minimum of 40 (or 10 under the more permissive threshold) transition events is required to define a state, allowing inclusion of 1 covariate in the transition model. When transitions are sufficient to include covariates in the model, at least 40 × *m* transition events are required. Covariate selection for each transition model, given the number of transitions, is described below. Follow-up time or care episode duration is defined as the interval from entry into the initial state (time 0) to entry into a terminal state.

**Figure 1.**
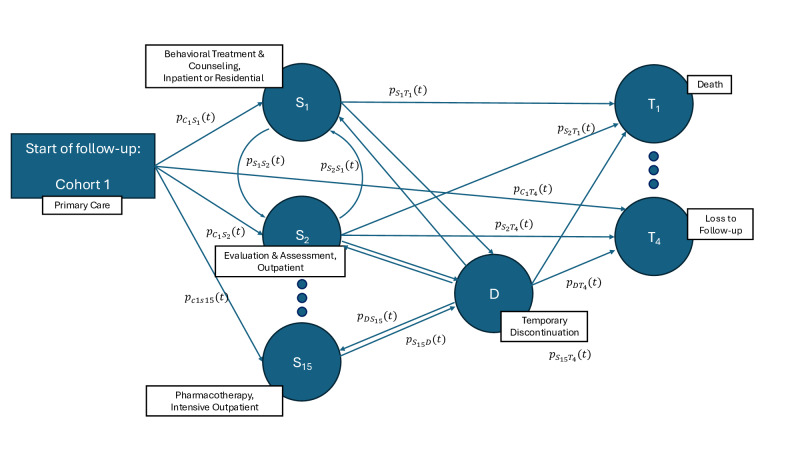
Graphical representation of the multistate model. Notation: Subcohort: [*C*_*1*_; care states (*S*_*1*_,...,*S*_*15*_); discontinuation of care: *D*; SUD remission state: *R*; terminal states: (*T*_1_,...,*T_4_*); *t*: time, *p*: probability] The 3 dots connecting the states indicate that the depicted relationships are possible for the other states not shown in this figure. p: probability; SUD: substance use disorder; t: time.

Figure 1 provides a graphical representation of a simplified MSM illustrating potential care pathways. In this example, patients who meet the inclusion criteria and are identified as having an incident SUD in primary care enter the initial state *C*_1_. Patients then transit into 1 of 15 care states (*S*_1_, ... ,*S*_15_) defined by a service type and setting. From these states, patients may transition to temporary discontinuation of care, *D*, or an SUD remission state, *R*. Transitions between the transient states (care states, temporary discontinuation, and SUD remission) are bidirectional, whereas any transition to a terminal state (*T*_1_, ..., *T*_4_) is unidirectional, reflecting that patients cannot leave terminal states once they are entered in MSMs. At the end of follow-up, patients occupy 1 of the 4 terminal states.

We will adopt a semi-Markov assumption [59] under which transition intensities depend on only the duration that patients spend time in the current state before transitioning. This framework allows transition intensities to vary over time rather than assuming constant transition intensities as in the Markov framework. Time is defined as the duration since entry into a state, with the clock reset to 0 upon entry into the cohort and each subsequent state. At any time since entry into the initial state, a patient occupies 1 of the 21 states (17 transient states and 4 terminal states). Movements between states *j* and *k* $\left(j,k=S_{1},\ldots ,S_{15},D,R,T_{1},\ldots ,T_{4}\right)$, are governed by a 21-by-21 matrix of transition intensities $q_{jk}(t,z(t))$, which is a function of time *t* and covariates z(t). Intensities capture the instantaneous probability of moving from state *j* to *k*.

Under the semi-Markov assumption, we will fit separate flexible parametric survival models [60,61] for each transition:

$\log\{q_{jk}(t,z(t))\}=s(\log(t)|\eta ,k_{0})+\beta^{T}z_{i}(t)$.

For simplicity, we omit transition-specific subscripts for the parameters $\left(\eta ,k_{0},\beta \right)$. In the model, $s(\log(t)|\eta ,k_{0})=\sum_{i=1}^{k_{0}}\eta_{i}B_{i}(\log(t))$ represents the log cumulative baseline intensity function, which we model flexibly using restricted cubic spline basis functions (also known as natural cubic splines) $B_{i}(\cdot )$, with corresponding coefficients $\eta_{i}$. The number of knots, $k_{0}$, determines the number of basis functions and thus the flexibility of the baseline intensity functions. For covariates *z*_*i*_(*t*), the coefficients β represent the log hazard ratios at time t. The unknown parameters to be estimated or selected from data are $\left(\eta ,k_{0},\beta \right)$. We will use the Akaike information criterion based on data to select the number of knots, with lower Akaike information criterion values indicating better model fit. The regression coefficient parameters (η,β) will be estimated by using the maximum likelihood approach for statistical inference [62,63]. For each transition, we will include prespecified covariates for adjustment when the number of transitions allows (at least 10 transition events per covariate), and if additional covariate selection is required, variables that are statistically significant at the 5% level based on Wald tests will be included [64]. The estimated transition intensities are transformed to a transition probability from states j to k at time t since entry into state j at time s (s<t, s and *t*>0), by integrating, $p_{jk}(s,t)=\exp\left\{-\int_{s}^{t}q_{jk}(u,z(u))\;du\right\}$.

Multiple incident SUD events may be observed within the same patient (see Quantitative Study Population). For example, a patient may have an incident SUD event, eventually experience a terminal state such as ceasing SUD care (eg, one who receives no SUD care for 194 days), and then experience a second incident SUD event. Including multiple incident events from the same patient within the same subcohort in the MSM framework may introduce within-patient correlation and reflect care pathways that differ from those of the initial episode. We will account for within-patient correlation using a sandwich variance estimator for the parameter estimates. The number of prior incident events will be included as a covariate or used as a stratification factor, depending on its distribution.

To assess model adequacy, we will compare spline-based estimates of the cumulative baseline hazard with nonparametric estimates derived from the Kaplan-Meier method for visual agreement. To evaluate the proportional hazards (time-independent effects) assumption, we will test for time-dependent effects by including interactions between covariates and spline functions of time. If these interaction terms are not statistically significant at the 5% level, they will be excluded from the final model.

Additionally, we assume that transition times are exactly observed. Lastly, it is assumed that the distribution of health care visit times is independent of transition probabilities for simplicity (noninformative visiting process). We will primarily use the R (R Foundation) package *flexsurv* to implement the MSMs and develop our own modules to transform the fitted results to answer research questions.

We will consider sensitivity analyses if sample size and other feasibility constraints permit. This includes stratified analyses by subgroups defined by the number of outpatient visits (eg, patients with more visits may have poorer health) [65,66] or clinically meaningful periods. For instance, impactful operational changes occurred due to COVID-19 mitigation restrictions (eg, reduced in-person care), so we will consider estimating models before, during, and after these restrictions were in place. Additionally, stratified analyses by SUD type will be considered to acknowledge that each substance may sometimes require unique forms of care, such as pharmacotherapy usage.

#### Aim 2. Generate Data-Driven Insights to Improve Care Delivery Using Estimated MSMs

We will apply the estimated MSM parameters from aim 1 to generate inferences that yield actionable data-driven insights to improve care delivery for SUD. As shown in Table 3, these inferences fall into three general categories: (1) examine SUD care states and terminal event rates, (2) identify empirically common care pathways, and (3) identify the extent to which guideline-concordant care is achieved.

**Table 3.** Summary of inferences from the multistate model and how they could be used by health systems to improve care for SUD^a^.

Key questions	Relevance to health systems	Analytic goal		
		Examine care states and terminal event rates	Identifies empirically common care pathways	Measure guideline-concordant care
How does the accessibility of reaching each care state vary?	Informs whether types of care are being reached at an expected rate, and whether resources are needed to support a given care type	✓		
How does the probability of occupying care states and terminal states vary over time?	Informs timing of interventions to address challenges in the delivery of SUD care	✓		
What are inefficient sequences of care following each initial state over time?	Identifies services or care patterns that need greater coordination (eg, multiple evaluations with delayed pharmacotherapy)		✓	
What is the contribution of each care state in achieving terminal states?	Prioritize SUD care types and inform places where specific SUD services are best delivered	✓		
What are the common (ie, most probable) sequences of SUD care following each initial state?	Helps health systems anticipate the needs of patients with SUD		✓	
How often and where is guideline-concordant care achieved?	Identifies care processes and locations to target for quality improvement			✓
How does the probability of occupying terminal states differ across patient subgroups?	Provides insights into whether outcomes differ for specific groups of patients who have incident SUD events in each setting	✓		
How do answers for the above key questions differ across patient characteristics?	Informs whether approaches to improve SUD care delivery should be tailored to different patient subgroups.	✓	✓	✓

a SUD: substance use disorder.

Table 4 summarizes estimates that can be obtained from fitted MSMs to generate our inferences and answer additional research questions. We will calculate point estimates and CIs and further characterize variation in output quantities across states graphically.

**Table 4.** Summary of multistate model output quantities.

Output	Description	Example question answered
Intensity matrix	Instantaneous probability of moving from 1 state to another	How likely is a transition from an initial primary care state to a behavioral treatment and counseling care state over a small incremental unit of time?
Transition probability matrix	Probability of moving from 1 state to another over a specific time duration	What is the probability of moving from an initial primary care state to a behavioral treatment and counseling care state within 14 days of SUD^a^ identification?
Hitting probability	Point estimate of the probability of ever reaching a state	What is the probability of ever reaching pharmacotherapy during a care episode?
Mean sojourn time	Mean length of time a patient stays in a state	On average, how long does a patient stay in the evaluation and assessment care state before transitioning to another state?
Restricted mean transition-free time	Mean length of time a patient spends in transient states before reaching a terminal state	What is the average time before a patient ceases SUD care after receiving pharmacotherapy?
Expected first passage time	Expected days until hitting a specific state of a set of states	How long until a patient receives SUD pharmacotherapy?
Expected number of visits	Expected number of times a state is reached between 2 given times	What is the predicted number of times behavioral treatment and counseling are reached in the first 30 days of a care episode?
Survival plot	Probability of not reaching a state, as a function of time	How is the probability of not ceasing SUD care after their index SUD event?

a SUD: substance use disorder.

How does the accessibility of each state vary? We will use the models to describe how the accessibility of each state varies by estimating the dynamics of transitioning between different types of care for SUD. We will report and characterize variation in (1) transition probabilities, (2) hitting probabilities, (3) mean sojourn time, (4) expected first passage time, and (5) expected number of visits (definitions in Table 4).

**Figure 2.**
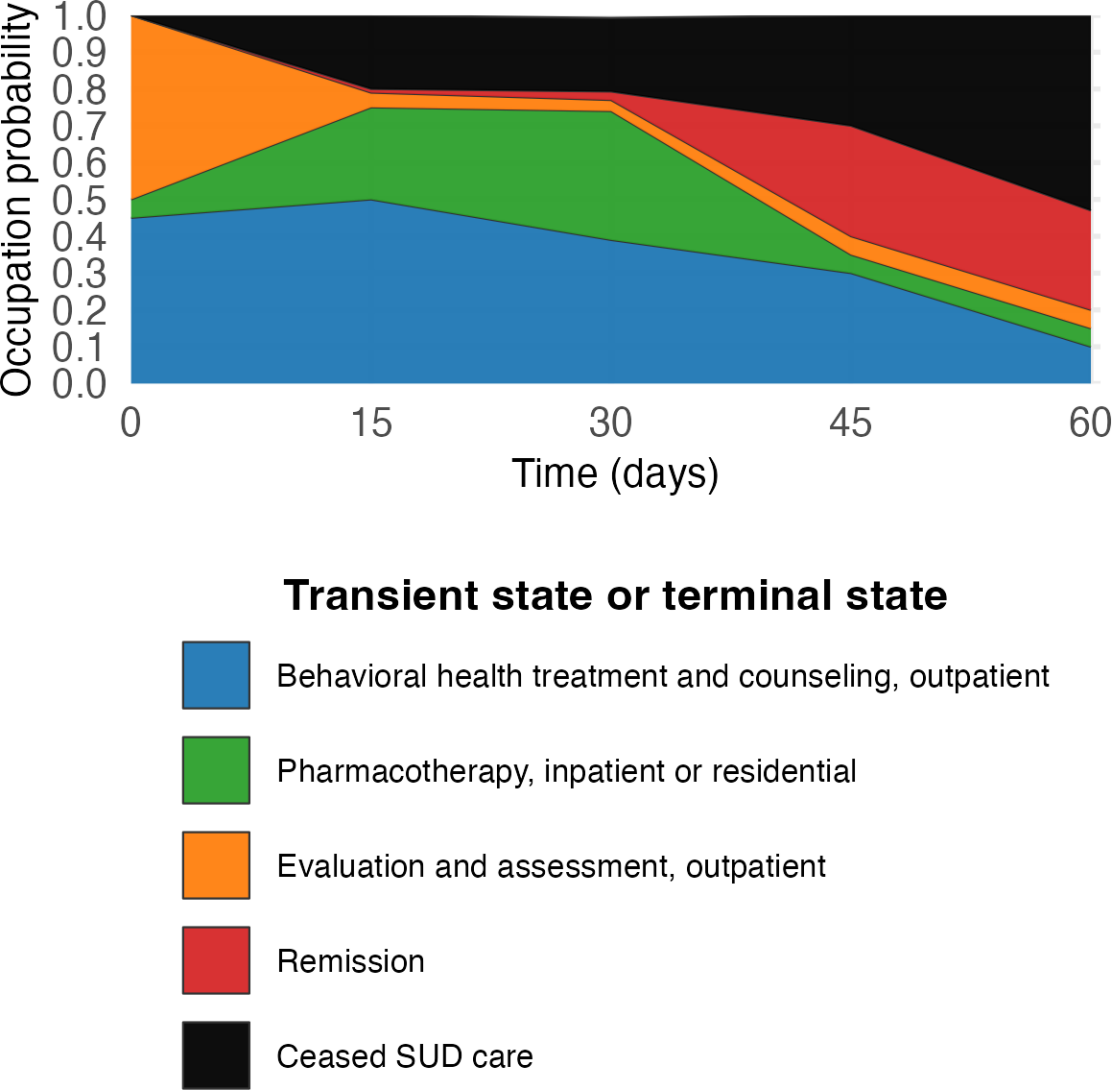
Stacked plot showing the probability of occupying specific care states or terminal over time (hypothetical). This hypothetical example shows the probability of ceasing SUD care increases steadily through day 15 and then plateaus through day 30, which could suggest a need for providing interventions to maintain engagement soon after a patient’s index SUD event. Over half of patients have ceased SUD care or have achieved SUD remission by day 45. SUD: substance use disorder.

How does the probability of occupying a given care state or terminal state vary over time? We will describe how the probability of occupying a state varies over time since cohort entry. To address this question, we will construct a stacked plot in which the y-axis represents the probability of being in each state, the x-axis shows time since cohort entry, and the stacked bands correspond to different states (Figure 2). Depicting the timing and probability of occupying states can help inform quality improvement interventions. For instance, by visualizing the time at which the probability of ceasing SUD care increases, it can help guide the timing of interventions to check for SUD care engagement and outreach to re-engage patients when needed.

**Figure 3.**
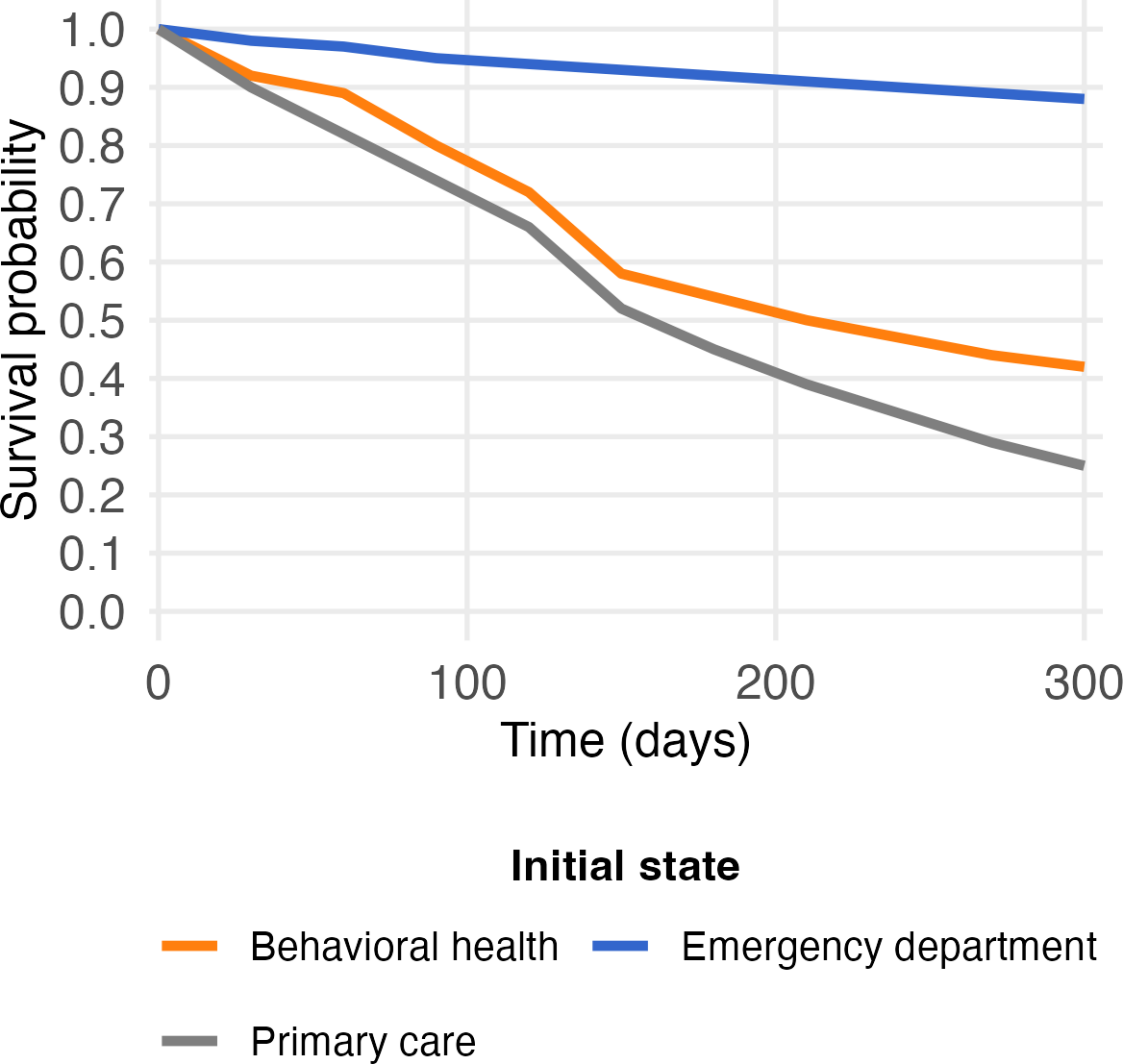
Transition-free (or survival) probability by initial access state (hypothetical). This hypothetical survival plot displays how the probability of not reaching a given state can be graphed as a function of the initial state. In this example, the probability of not reaching behavioral treatment and counseling in an outpatient setting (not shown) is markedly higher when the incident substance use disorder event occurs in the emergency department setting, when compared to primary care or behavioral health settings. Potentially, this is suggestive of inefficiencies in referring people from emergency department settings to behavioral treatment and counseling in an outpatient setting.

What are inefficient sequences of SUD care following each initial state over time? Several inferences from the MSM may be a signal for inefficient SUD care. First, prolonged time in some states, such as inpatient care, may reflect challenges in coordinating care with services following an inpatient stay. We will thus identify states with relatively long mean sojourn time [67]. Second, care states with a low probability of being reached, or that take a long time before they are reached, may reflect barriers to care such as long wait times. To identify these care types, we will explore states with long expected first passage times [68] and low hitting probabilities [67]. We will also construct survival plots to display how the probability of starting in an initial access point (eg, index SUD event in primary care) and not reaching a target care state changes over time. For example, Figure 3 displays the probability of not reaching a target care state (eg, behavioral treatment and counseling in an outpatient setting) from different initial states. In this example, the probability of not reaching behavioral treatment and counseling in an outpatient setting following entry into the ED cohort is markedly higher than the corresponding probabilities following entry into the primary care or MHAC cohorts, suggestive of referral inefficiencies in ED services.

**Figure 4.**
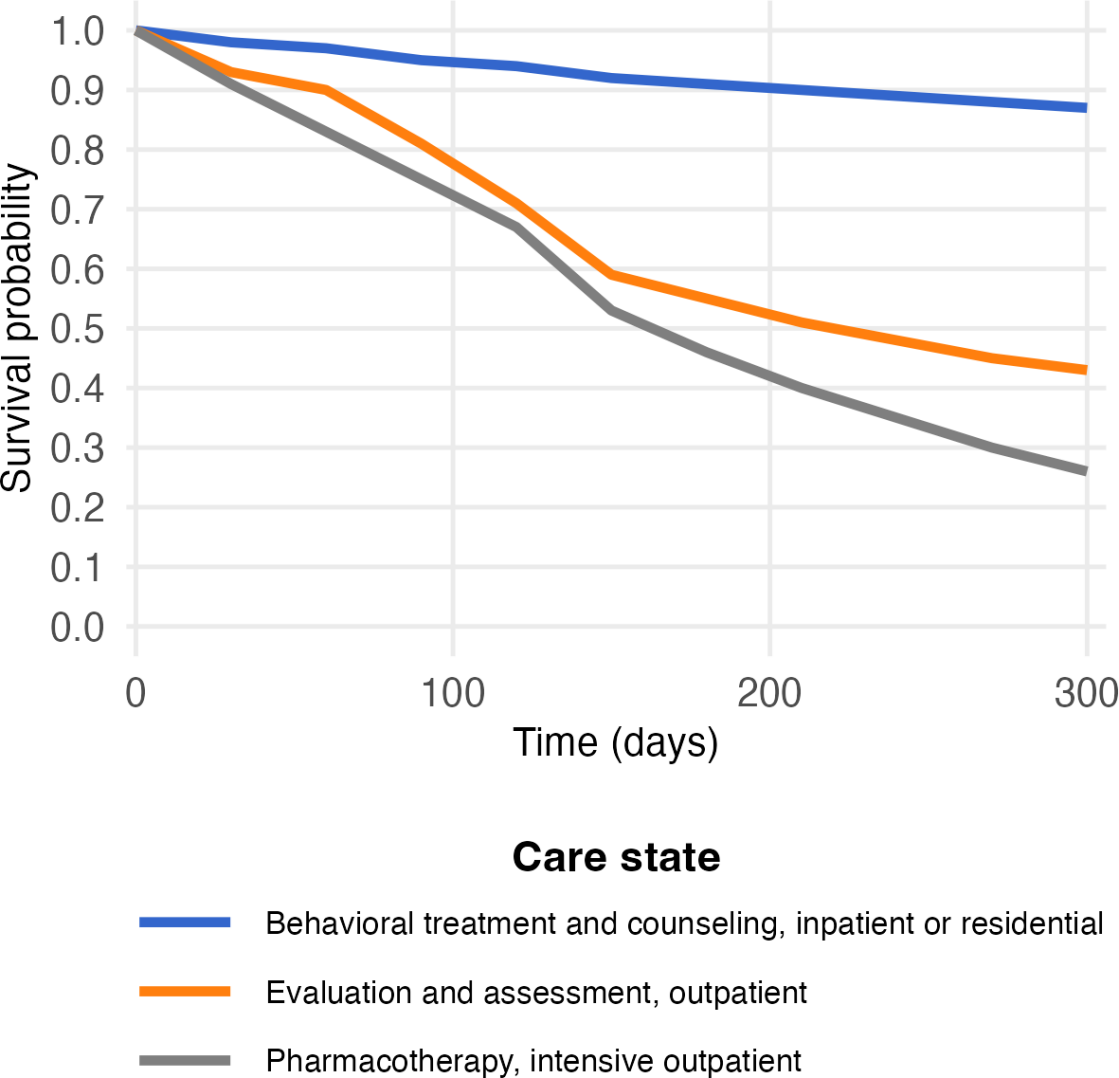
Transition-free (or survival) probability by care state (hypothetical). This hypothetical survival plot displays how the probability of not reaching a given terminal state can be graphed as a function of care states. In this example, the probability of not transitioning to the state of ceasing substance use disorder (SUD) care (not shown) is markedly higher for patients receiving behavioral treatment and counseling in inpatient or residential settings, when compared to patients receiving evaluation and assessment in an outpatient setting or pharmacotherapy in an intensive outpatient setting. Potentially, this can inform targeted efforts such as enhancing care coordination or improving staff levels in specific settings or for specific treatment modalities to increase retention in SUD care.

What is the contribution of care states in achieving terminal states? We will calculate restricted mean survival times (RMSTs) [69] between a given SUD care state and each terminal state. An example of RMST quantities between select care states and a terminal state is presented in Table 5. The RMST captures the average terminal-event-free time, which in this study reflects time before reaching a terminal state, calculated as the area under the terminal-event-free curve over a prespecified time interval since the entry to the given care state. Figure 4 presents an example of terminal-event-free curve estimates using states from Figure 1. In this example, care types that produce lower RMSTs are those more likely to end in ceasing SUD care. Identifying these care types can inform targeted efforts, such as enhancing care coordination or improving staff levels in specific settings or for patients who receive certain treatment modalities to increase retention in SUD care.

**Table 5.** Restricted mean survival times (RMST) from selected care types to ceasing care.

Substance use disorder (SUD) care state	Follow-up time (days)		
	30	60	90
Behavioral treatment and counseling, outpatient	μ₁,₄(30)^a^	μ₁,₄(60)	μ₁,₄(90)
Evaluation and assessment, intensive outpatient treatment	μ₂,₄(30)	μ₂,₄(60)	μ₂,₄(90)
Pharmacotherapy, outpatient	μ₃,₄(30)	μ₃,₄(60)	μ₃,₄(90)

a 𝜇_i_,_𝑗_(𝑡_𝑜_) is the restricted mean survival times from state i to state j over time interval 𝑡_𝑜_, where i=1 (behavioral health counseling, outpatient), 2 (evaluation and management, intensive outpatient treatment), and 3 (pharmacotherapy, outpatient); j=4 (ceasing substance use disorder care).

What are common sequences of care following each initial state? Care pathways consist of a sequence of transitions across care types and settings. To characterize these sequences, we will calculate the probabilities of all identified pathways starting from a given initial state in Table 1, proceeding through multiple care states, and ending in a specific terminal state. We will estimate probabilities of sequences of varying length. The length of sequences will be informed by descriptive analysis examining the mean and median number of transitions among subgroups of patients achieving terminal outcomes in Table 2. Sequence probabilities will be estimated using the fitted MSMs under hypothetical scenarios, such as repeated cycling between care and care-discontinuation and specified transition timings (see Multimedia Appendix 2).

How often and where is guideline-concordant care achieved? We will analyze several health care quality measures, including those published by the National Committee on Quality Assurance [36,70,71] and others [72,73]. These monitor initiation of care, or the receipt of prespecified health services following an incident SUD event; engagement in care, defined as having a minimum number of qualifying services after initiating care; and adequacy of treatment, which captures whether quantity or duration of treatment over time is sufficient. Achievement of these measures can be ascertained from the states in the MSM model. Multimedia Appendix 1 contains details about how we will operationalize these quality measures using estimates from the fitted MSMs. For example, a National Committee on Quality Assurance quality measure monitors follow-up for patients who have an acute care encounter for SUD. This measure monitors the percentage of patients who obtain follow-up SUD care after an ED visit over 7-day and 30-day timeframes. Thus, among patients in the ED and hospitalizations subcohort whose incident SUD event is in an ED setting, we will estimate transition probabilities capturing the likelihood of transitioning from the initial state to any SUD care state within 7-day or 30-day. We will estimate measures reflecting care engagement and adequacy of treatment by estimating the expected length of time in a pharmacotherapy state or the expected number of visits to qualifying states over the duration of time specified in Multimedia Appendix 1.

How does the probability of occupying terminal states differ across patient subgroups? Within study subcohorts, we will conduct descriptive analysis comparing occurrences of terminal states across patient subgroups. For instance, we will compare terminal states at selected time points (eg, 1-year after cohort entry) across mutually exclusive subgroups defined by patient SUD type (eg, sole opioid, alcohol, or cannabis use disorder vs multiple SUDs). Comparisons of terminal states will help provide insights into whether outcomes differ for specific groups of patients who have incident SUD events in each setting [28].

How do answers for the above key questions differ across patient characteristics? First, overall quantities will be estimated to address the key questions in Table 4. Then, Cox proportional hazards models will be fit for each transition intensity to assess associations between patient characteristics, such as demographics, SUD type and severity, mental health diagnosis, socioeconomic status, and transition intensities [9]. Using the fitted model, the quantities required to address the questions in Table 4 will be calculated as a function of patient characteristics included in the transition models.

#### Aim 3. Observe and Explore Patient and Clinician Experiences With Care Transitions Across Common Care Pathways Using Qualitative Research Methods

Aim 3 includes observations and interviews to explore the context for SUD care decisions and experiences with SUD care transitions. We will perform direct observations [74,75] of patient-clinician encounters during salient SUD visits, such as behavioral health intake visits. Witnessing and documenting these interactions will provide key information on decision-making regarding various clinical scenarios. We will also conduct semistructured in-depth interviews with a broad sample of patients and clinicians to collect views, preferences, and needs of individuals engaged in different care pathways. Observations and interviews will be concurrent to inform ongoing data collection and confirm data sufficiency [76]. We will draw on phenomenological [77] and ethnographic theory [78] to explore emic [79], or insider perspectives on care transitions.

The quantitative and qualitative components synergize in 2 ways: first, by using an explanatory sequential sampling mixed methods design [80] whereby qualitative interview participants are purposely sampled from potentially meaningfully rich subgroups of patients who have embarked on specific care pathways identified in the quantitative population. Second, by integrating quantitative and qualitative data in the analysis phase. Specifically, we will reapply the inclusion criteria for aims 1 and 2 to identify patients who were recently identified to start an episode of SUD care at the time of qualitative data collection. We will then match the patient’s observed sequence of care to model-identified pathways estimated in aim 1. We will then purposively sample [81] patients from six pathways: (1) two of the overall most common care pathways, (2) two of the most common care pathways that contain guideline-concordant care, and (3) two of the most common care pathways that have a high probability of reaching negative terminal states (eg, death). We will be open to the emergence of other important care patterns to guide our sample selection, and we may consider other important factors, such as SUD type and/or severity, when defining these groups. We will sample approximately 3‐5 patients from each pathway (estimated to be 35 patients) and ensure representation across demographic groups and those with different SUD types.

For the patient interviews, a semistructured interview guide will include three main topics: (1) care choices and constraints at critical junctures (eg, referral to specialty services), (2) care alignment with patient needs and preferences (eg, care frequency and location), and (3) clinical and care quality outcomes (eg, treatment adequacy). Interviews will use a modified form of free listing [82-84], an ethnographic technique increasingly used in health research [85-87] to elicit insider views and conceptions of a cultural or experiential domain (eg, process of obtaining SUD care). We will ask patients to rapidly say or write words that come to mind when thinking of an experience (eg, patient decision-making). Their words are assumed to indicate salient constructs within a domain (eg, treatment options) common to people who share the experience and help reveal the conceptual boundaries of the domain of interest. This provides a foundation for further inquiry into health-related experiences, attitudes, beliefs, and behaviors [88]. We will query how patients conceive of and define care journeys, potential choices, and instructions from clinicians. We will draw from the Chronic Care Model [29] to inquire how the free-listed words affected the ability to achieve outcomes, and how clinicians and systems can better support care transitions.

For clinician interviews, we will interview employees of KPWA or external organizations in the contracted care network who help patients make SUD care decisions in primary care, ED, urgent care, behavioral health, and specialty SUD care. We will oversample clinicians who provide care in roles that are consistent with the model-identified common care pathways and ensure representation across clinician disciplines and care settings. We will recruit clinicians over email to arrange a video interview [89]. We anticipate recruiting approximately 25 clinicians. Guided by the Chronic Care Model, interviews will focus on decision-making and system- and practice-level factors affecting quality and coordination of SUD services. Questions will consider practice guidelines and information systems used to determine treatment plans, and how delivery system design affects referral choices. Per relevant literature, we will probe barriers and facilitators to care coordination (eg, program capacity), patient engagement, and meeting care needs. We will query beliefs and experiences tied to the role clinicians play in enhancing care continuity, and how roles can be strengthened [90]. All interviews will be conducted by phone or videoconference platform, recorded, and professionally transcribed.

We will also conduct patient-clinician observations during visits where SUD treatment planning takes place, such as scheduled SUD intake evaluations, primary care visits that follow ED or inpatient discharge, addiction psychiatry consultation, and buprenorphine induction. We will conduct up to 20 observations (≈5 per visit type), monitoring for data saturation, to capture nuances of the patient experience at critical transition points [91]. We will contact clinicians via email to invite them to have their visits observed. When a clinician agrees, a researcher will be available when the clinician begins the scheduled video or phone-based session. The clinician will ask the patient if a researcher could join to observe. If the patient agrees, the researcher will join the video call, invite the patient to participate in study observations, and obtain verbal informed consent. A semistructured observation template will be developed for taking field notes to document the context in which care options are considered and decided upon, and assess multilevel factors affecting care planning and pathways pursued. The template will be organized by the Chronic Care Model constructs and allow for unstructured notes and reflections. After each data collection (interview or observation), qualitative researchers complete a structured memo to identify data gaps and challenge assumptions. Prompts such as “what surprised you” and “what is unanswered” help the researcher reflect on initial assumptions and reveal data gaps to explore. After 5 data collections, memos are summarized and discussed to monitor data quality and completeness. Physician and social work investigators, partners, and consultants will join debriefs as data collection unfolds to identify new questions to be pursued.

A total of 2 researchers will analyze all qualitative data using thematic analysis [92]. We will code transcripts and observation notes using deductive and inductive codes [93]. The initial code list will be developed from interview and observation guide questions and prompts and Chronic Care Model domains. Inductive codes will be developed from reading transcripts and notes. Further, 2 researchers will read the same subset of transcripts to derive emergent codes, discussing and resolving discrepancies by consensus. We will combine all the codes, refine the code list via an iterative process, and rely on the Atlas Ti qualitative platform [94] to apply the final codes to transcripts and observation notes. Lastly, we will examine the coded text to highlight key themes and subthemes and use coding memos to summarize and further refine themes. Coding memos will form the basis for disseminating study results. We estimate that our patient and clinician samples will ensure the analytic depth, rigor, and trustworthiness of the findings, but we will monitor thematic saturation during analysis and recruit additional participants if needed [91,95]. We will triangulate interview and observation data to interrogate qualitative findings [96,97] and reach robust conclusions. Then we will integrate qualitative and quantitative findings by constructing a joint display of key qualitative themes and salient estimated quantities [80]. The joint display will highlight patterns, reveal concordant or discordant findings, and expand overall interpretation [98].

## Results

This study was funded in June 2025. Aims 1 and 2 received institutional review board approval on July 17, 2025. We expect preliminary data collection for the quantitative analyses to be complete by May 2026, and final data collection will be complete by November 2027. We expect to submit institutional review board approval for aim 3 by May 2027. We expect that data collection for the qualitative aim will be complete by November 2029.

## Discussion

### Study Summary

This study will be the first to apply MSMs to evaluate the complexity of SUD service provision across any large integrated health care system. Quantitative findings will provide an essential foundation for understanding when and how patients obtain SUD treatment in real-world settings. Qualitative findings will elucidate why and how patients experience pathways, including the contextual, system-level, and patient-level factors that contribute to delays, disengagement, or receipt of evidence-based care. Health systems urgently need solutions that address treatment gaps for an increasingly prevalent and devastating condition. While this study is exploratory in nature, findings from this study will yield numerous real-world insights that are directly translatable to clinicians (eg, providing information about common care pathways among various treatment options in integrated care settings), health systems (eg, identifying where patients may become lost in the system and fall out of the treatment process), and policymakers (eg, informing policies that support coordination across different treatment clinicians or agencies spanning the continuum of care).

### Limitations

Practical limitations require us to set an achievable scope for the analyses; we chose care states and terminal states that are measurable and salient to health systems. However, we recognize that others may also be important. Second, while we can account for patient characteristics in the estimation of MSMs, inferences do not have a causal interpretation as in randomized trials. Third, this study relies on administrative data (EHR and claims), which reflect services and conditions that are recorded for clinical or billing purposes and may not capture care received outside the system (eg, private pay), informal services (eg, mutual aid), or unidentified needs. Fourth, the use of administrative data could lead to misclassification. For example, while substance use measures have excellent psychometric properties, some studies have pointed to underreporting of substance use among some patients [99]. Fifth, the quantitative analyses focus on a single health system in Washington State, KPWA, that primarily serves people with employer-based insurance or Medicare. Findings should not be taken to represent all health care settings. Finally, qualitative analyses will examine the experiences and perspectives of a subsample of patients with SUD and their clinicians. Insights from these analyses may not generalize to all patients, an inherent but manageable limitation to qualitative research.

### Conclusions

This protocol outlines the design of a mixed-methods study that draws upon powerful quantitative and qualitative methods not typically used in health services research. MSM models will represent a new scientific innovation that comprehensively summarizes the many possible ways that patients may progress through the health care system during an SUD treatment episode, addressing a significant and substantial scientific evidence gap. Integration of model-derived pathways with observations and qualitative interviews will provide critical insight into the decision-making processes and contextual factors that shape care pathways and transitions. Together, this exploratory research provides the framework for generating inferences to inform health system improvements to increase initiation, engagement, accessibility, and coordination of SUD care.

## Supplementary material

10.2196/93043 Multimedia Appendix 1 Operationalization of guideline-concordant care via substance use disorder care quality measures.

10.2196/93043 Multimedia Appendix 2 Multistate model estimation quantities: examples and calculation details.

## Abbreviations

AHRQ: Agency for Healthcare Research and Quality
CFR: Code of Federal Regulations
*DSM-5*: *Diagnostic and Statistical Manual of Mental Disorders, Fifth Edition*
ED: emergency department
EHR: electronic health record
HEDIS: Healthcare Effectiveness Data and Information Set
HIPAA: Health Insurance Portability and Accountability Act
*ICD*: *International Classification of Diseases*
*ICD-10*: *International Classification of Diseases, Tenth Revision, Clinical Modification*
IDS: internal delivery system
KP: Kaiser Permanente
KPWA: Kaiser Permanente Washington
MHAC: MHAC
MSM: multistate model
RMST: restricted mean survival time
SDOH: social determinants of health
SUD: substance use disorder
