# Supplementary material for: Using Multistate Models and Qualitative Interviews to Comprehensively Characterize Substance Use Disorder Care Transitions in a US Health Care System: Protocol for a Mixed-Methods Study
Source: JMIR Res Protoc. 2026 May 28;15:e93043. doi: 10.2196/93043 (PMC13218565; doi:10.2196/93043)
Supplement: Multimedia Appendix 1 — Operationalization of guideline-concordant care via substance use disorder care quality measures. [file resprot-v15-e93043-s001.docx]

| Table S1. Preliminary operational definitions for measuring guideline-concordant care for substance use disorder (SUD) using estimates from fitted multi-state models. | | |
| --- | --- | --- |
| Measure | **Definition** | **Rationale** |
| *SUD Care Initiation and Engagement* | | |
| Engagement in SUD Care | The probability that a patient has two or more visits to any SUD care state within approximately one month of initiating SUD care [1,2] | Early and regular SUD treatment is linked to improved outcomes for patients with SUDs. |
| Follow-Up After emergency department (ED) visits for SUD | The probability of making a visit to any SUD care state within 7 days and within 30 days of entering the emergency department or hospitalization cohort [1,3] | Ensures patients receive follow-up after a high-risk substance use event, helping ensure their care is coordinated. |
| Use of Pharmacotherapy for  Opioid or Alcohol Use Disorder | Separately among patients who enter the study cohort with OUD or AUD, the probability that they received medication for their condition before reaching a terminal state | Patients with receiving medications have lower mortality than those not receiving medication. |
| *Adequacy of Treatment Measures* | | |
| Continuity of Pharmacotherapy for Opioid or Alcohol Disorder | Among patients who received pharmacotherapy, the probably of having a cumulative 180 days of medication dispensed w/ ≤7-day gap, starting on the first day that the patient received pharmacotherapy [4] | Pharmacotherapy persistence is associated with survival and better outcomes. |
| Adequate Psychotherapy Dose | Among patients who received behavioral treatment and counseling, the probability of having four or more behavioral treatment and counseling visits in any setting within 8 weeks of the first behavioral treatment and counseling visit [1,5,6] | Retention in behavioral treatment such as psychotherapy results in improved outcomes |

1. Schmidt EM, Liu P, Combs A, Trafton J, Asch S, Harris AHS. Surveying the Landscape of Quality-of-Care Measures for Mental and Substance Use Disorders. Psychiatr Serv 2022 Aug 1;73(8):880–888. doi: 10.1176/appi.ps.202000913

2. Harris AHS, Bowe T, Finney JW, Humphreys K. HEDIS Initiation and Engagement Quality Measures of Substance Use Disorder Care: Impact of Setting and Health Care Specialty. Popul Health Manag Mary Ann Liebert, Inc., publishers; 2009 Aug 1;12(4):191–196. doi: 10.1089/pop.2008.0028

3. National Committee for Quality Assurance. Follow-Up After High-Intensity Care for Substance Use Disorder (FUI). Available from: https://www.ncqa.org/hedis/measures/follow-up-after-high-intensity-care-for-substance-use-disorder/ [accessed May 6, 2024]

4. Williams AR, Mauro CM, Feng T, Wilson A, Cruz A, Olfson M, Crystal S, Samples H, Chiodo L. Performance Measurement for Opioid Use Disorder Medication Treatment and Care Retention. AJP 2023 June 1;180(6):454–457. doi: 10.1176/appi.ajp.20220456

5. Grekin R, Bohnert KM, Grau PP, Ganoczy D, Sripada RK. Rates and Predictors of Psychotherapy Receipt Among U.S. Veterans with Comorbid Posttraumatic Stress Disorder and Substance Use Disorders. Drug Alcohol Depend Rep 2021 Dec;1:100010. PMID:36843911

6. D.33 SAMPLE CBOC DASHBOARD, CURRENT PERFORMANCE MEASURES, AND EXTERNAL PEER REVIEW PROGRAM (EPRP) INFORMATION. Available from: https://www.vendorportal.ecms.va.gov/FBODocumentServer/DocumentServer.aspx?DocumentId=4631855&FileName=36C24218R0169-059.pdf [accessed May 24, 2024]
