# Supplementary material for: Using Multistate Models and Qualitative Interviews to Comprehensively Characterize Substance Use Disorder Care Transitions in a US Health Care System: Protocol for a Mixed-Methods Study
Source: JMIR Res Protoc. 2026 May 28;15:e93043. doi: 10.2196/93043 (PMC13218565; doi:10.2196/93043)
Supplement: Multimedia Appendix 2 — Multistate model estimation quantities: examples and calculation details. [file resprot-v15-e93043-s002.docx]

Calculations for estimating sequences probabilities to derive common sequences of care following each initial access point.

Consider a sequence and each state in the sequence is indexed by $i$. Denote the probability of transitioning from state $S_{i}$ to $S_{i+1}$ is $p_{S_{i}, S_{i+1}}$. The probability of a sequence is given by $\prod_{i=1}^{n-1} p_{S_{i}, S_{i+1}}(t_{i},t_{i+1})$ for ${0<t}_{i}<t_{i+1}$ and $i=1,\ldots, n-1$, where $p_{S_{i}, S_{i+1}}\left( t_{i},t_{i+1} \right)$ denotes the transition probability from state $S_{i}$at time $t_{i}$ to state $S_{i+1}$at time $t_{i+1}$. In a continuous time setting, care time units are used. For example, suppose that the unit of pharmacotherapy prescription is 1 week, and a patient occupies the pharmacotherapy state for 3 weeks, then this can be represented as 3 recursions in discrete time framework.
